# Developing an Automatic Color Determination Procedure for the Quality Assessment of Mangos (*Mangifera indica*) Using a CCD Camera and Color Standards

**DOI:** 10.3390/foods9111709

**Published:** 2020-11-21

**Authors:** Khanitta Ratprakhon, Werner Neubauer, Katharina Riehn, Jan Fritsche, Sascha Rohn

**Affiliations:** 1 Department of Nutrition and Home Economics, Faculty of Life Sciences, University of Applied Sciences Hamburg, Ulmenliet 20, 20133 Hamburg, Germany; khanitta.ratprakhon@haw-hamburg.de (K.R.); katharina.riehn@haw-hamburg.de (K.R.); jan.fritsche@mri.bund.de (J.F.); 2 Institute for Food Chemistry, Hamburg School of Food Science, Universität Hamburg, Grindelallee 117, 20146 Hamburg, Germany; 3 win ing.-Büro Werner Neubauer, Paradiesweg 4, 96148 Baunach, Germany; wn@fpga-design.de; 4 Department of Food Chemistry and Analysis, Institute of Food Technology and Food Chemistry, Technische Universität Berlin, TIB 4/3-1, Gustav-Meyer-Allee 25, 13355 Berlin, Germany

**Keywords:** mango color, CCD camera, computer vision system, NCS color standard

## Abstract

Color is one of the key sensory characteristics in the evaluation of the quality of mangos (*Mangifera indica*) especially with regard to determining the optimal level of ripeness. However, an objective color determination of entire fruits can be a challenging task. Conventional evaluation methods such as colorimetric or spectrophotometric procedures are primarily limited to a homogenous distribution of the color. Accordingly, a direct assessment of the mango quality with regard to color requires more pronounced color determination procedures. In this study, the color of the peel and the pulp of the mango cultivars “Nam Dokmai”, “Mahachanok”, and “Kent” was evaluated and categorized into various levels of ripeness using a charge-coupled device (CCD) camera in combination with a computer vision system and color standards. The color evaluation process is based on a transformation of the RGB (red, green, and blue) color space values into the HSI (hue, saturation, and intensity) color system and the Natural Color Standard (NCS). The results showed that for pulp color codes, 0560-Y20R and 0560-Y40R can be used as appropriate indicators for the ripeness of the cultivars “Nam Dokmai” and “Mahachanok”. The peels of these two mango cultivars present two distinct colors (1050-Y40R and 1060-Y40R), which can be used to determine the fruit maturity during the post-ripening process. However, in the case of the cultivar “Kent”, peel color detection was not an applicable approach for determining ripeness; thus, the determination of the pulp color with the color code 0550-Y20R gave promising results.

## 1. Introduction

Color is an important factor for the quality assessment of fresh produce, as it can be used to estimate the ripeness of fruits and vegetables. A survey of the existing literature revealed, for example, that Khairunniza-Bejo et al. (2014) used color to determine internal attributes, like sweetness [1]. Jha et al. (2007), as well as Nambi et al. (2015), used color to study internal properties, such as fruit maturity [2,3].

According to Nagle et al. (2016), chlorophylls, carotenoids, and anthocyanins are responsible for the green, yellow, and red colors of fruit and vegetables, respectively, also representing the key pigments in mango (*Mangifera indica*) maturation [4]. Hence, these natural pigments might be applicable for the visual color assessment of mangos. In addition, changes in mango peel and pulp color reflect their maturity development during the post-ripening process [5,6]. Typically, mangos are regarded as fully ripe when they change to a juicy and soft texture, with a sweet taste and rich aroma, and fully developed coloring depending on cultivars [7]. This behavior can also be found in many other (climacteric) fruits, such as blueberries, where color significantly changes from slightly green to deep purple tones [8]. For color measurement, spectrophotometry is a technique widely used to determine produce color by measuring the spectral distribution of transmittance using a sample’s reflection [9]. Being traditionally non-destructive instruments, colorimeters are extensively used in the fruit industry for measuring the color of fruits [10]. In this context, uniform measurements achieved with the so-called Commission Internationale d’Eclairage (CIE) L*a*b* (CIELAB) color space, as standardization and visual assessment, can be very precise [11]. Generally, CIE L*a*b* (CIELAB) is the most complete color model, being used conventionally for describing colors visible to the human eye. It was developed for this specific purpose by the Commission Internationale d’Eclairage (CIE). However, there are further models that seem to be easier to handle and seem to be more intuitive when color changes. Some color models and methods can also be beneficial, when measurements and evaluations have to be carried out directly on-site (e.g., for monitoring fruit ripeness). Another important aspect to consider is that the entire fruit needs to be characterized [12]. In addition, a number of fruits must be assessed so that they can be representative of the entire field. These aspects can be limited when using colorimeters and CIELAB [12]. However, using colorimetric measurements and CIELAB is often limited with regard to sampling area compared to the size of the fruit [13]. Consequently, modified two-dimensional color imaging is required to overcome this limitation. Even more easy to apply, photons reflected from the fruit’s skin can be detected and converted to electric signals, e.g., by a charge-coupled device (CCD) camera [12]. However, alternative techniques and models have to be developed and tested in order to determine the heterogeneous color distribution of mangos, especially when the uneven shape of the mango fruit has to be considered as well and measurements should be done with easy-to-use equipment.

The importance of computer vision systems in the quality assessment of food has steadily increased in recent years. The biggest advantage, when compared to traditional methods, is that each pixel of the entire detected surface is included in the analysis, resulting in complex color modeling approaches assisted by multivariate statistical methods [2]. Image capturing and image processing are the main components of machine vision systems. Due to their low noise levels, high sensitivity, and great dynamic range [14], CCD cameras seem promising for mango color evaluation. According to most of the literature surveyed, computer vision systems are mostly used for automatic mango fruit grading based on color systems [4,15,16,17,18,19].

The pre-processing pixel analysis used in this work for converting input images into output images is based on a transformation of RGB values into the HSI color system that defines hue (H), saturation (S), and intensity (I). The RGB color space is a three-dimensional color system that constructs all colors from the combination of the colors red, green, and blue. Similarly to the CIELAB color space, it is possible to analyze fruit ripeness through RGB values. This system can be regarded as a cubic model, where *red*, *green*, and *blue* are the key colors of the RGB color space on the different axes. This model has serious disadvantages when someone wants to perform different types of processing of the images such as enhancement, segmentation, or classification. To reduce such limitations, alternative three-dimensional models have been developed that separate the color from the lighting information. For example, the HSI space further considers *hue*, *saturation*, and *intensity* of a color. This model has been described as suitable for food technological research questions such as the evaluation of the quality of fruits [14,20,21]. With appropriate calibration, it is possible to process images with changes in light, as the ambient colors can be differentiated from one another by the color tone component and certain calibration sets (e.g., with color cards), with which external influencing factors can be minimized [14]. The use of such references is easy to handle and the influence of even more complex conditions such as lighting changes in the field or camera-dependent parameters can be reduced. Comparatively, CIELAB encounters the out-of-gamut problem. It depends on the shape of the 3D color gamut and which color space (CIELAB vs. RGB/HSI) is easier to use and assess (e.g., for estimating thresholds). Consequently, CIELAB is not necessarily superior to HSI and vice versa. When cylinder coordinates are needed, HSI seems to be easier [14]. However, this model has been designed to match human intuition [20]. It seems to also have advantages in image processing, such as color image enhancement, segmentation, fusion, color-based object detection, recognition, and traffic signal detection [21].

As in the RGB system (estimated with a CCD camera), a digital image in the HSI space also consists of values of hue, saturation, and intensity/brightness. RGB and HSI values can be converted into each value by data conversion. However, initially, before the RGB-to-HSI conversion, it is necessary to normalize the R, G, and B components of a pixel in a color image from the range of (0, 255) to (0, 1), which can be performed automatically by free as well as commercially available software [22]. 

The HSI color system is commonly used in the food industry, with *hue* defining perceived color, *saturation* measuring color density, and *intensity* representing color brightness or illuminance [14]. The stabilization of illuminance values is of particular importance for obtaining precise results. Because of the many possible variants, the application of the HSI color system is comparable to the human visual perception of food surfaces [23]. Blasco et al. (2007) also used this color space to study quality defects of citrus fruits [24]. Similarly, Abdullah et al. (2006) converted RGB values into HSI values for the classification of starfruits (*Averrhoa carambola*) into four levels of maturity [25].

In this work, the idea and concept of developing the computer vision method were derived from the German national standard DIN EN 60350, which sets the standards for measuring the performance of particular household electric cooking appliances such as ranges, ovens, steam ovens, and grills [26]. It describes the color brown for determining the degree of food browning using a computer vision system. However, there is still a lack of studies connecting color evaluation to a standard color system or other well-known color mappings.

Developed in Sweden, the Natural Color Standard System^®^ (NCS) has continuously become the international color standard most commonly used in the food industry. It classifies colors into six types, labeling them as elementary colors, namely whiteness (W), blackness (S), redness (R), yellowness (Y), greenness (G), and blueness (B), all of which are perceived by the human sense of color. Color is specified by three main parameters expressed in percentages: blackness, chromaticness, and hue. Whiteness is determined by subtracting the sum of blackness and chromaticness from 100% [27]. The application of a color standard ensures increased stability, consistency, and applicability in measuring systems [28]. A display of color can be presented by using a color map or color code standard. Instead of only indicating a color value, this method allows for a direct color detection. 

This study aimed at developing a visual RGB/HSI-based measurement and quantification method for the automated color assessment of the peel and/or the pulp of mangos measured at various stages of maturity. Moreover, the color spectrum of mango ripeness was coded by means of color standards in order to simplify the evaluation. In more detail, the study aimed at providing a comprehensive color evaluation of mangos using a computer vision system for both homogeneous- and heterogeneous-colored mangos using a new method that combines the application of a color system and a color code standard. The mango cultivar “Nam Dokmai” was selected to represent a homogenously colored mango, as its peel color changes from light green-yellow to gold-yellow, while the mango cultivars “Mahachanok” and “Kent” represent heterogeneously colored varieties, providing a mix of red and green colors on their surface.

## 2. Materials and Methods

### 2.1. Samples

**Figure 1:**
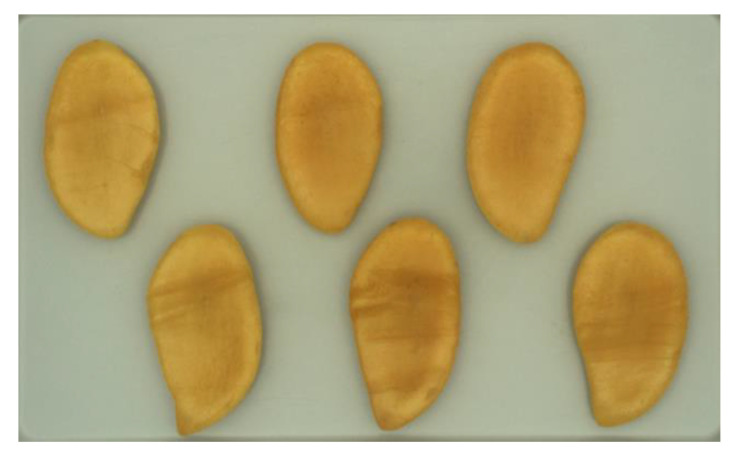
Slices of “Nam Dokmai” mango for evaluating the pulp color in the computer vision system.

The mango cultivars “Nam Dokmai” (NDM) and “Mahachanok” (MHC) used in this experiment were harvested 110 days (for NDM) or 98–100 days (MHC) after full bloom, respectively. They were sent immediately from Thailand to Germany by airfreight, while the cv. “Kent”, which can be purchased at the wholesale market in Hamburg, Germany, were initially transported from Peru to Hamburg, Germany, by ship. All samples were allowed to post-ripen in a climacteric chamber (Typ SB222/500, 300 L, Weiss Umwelttechnik GmbH, Reiskirchen, Germany) with temperature, humidity, and ethylene gas regulated at 30 °C, 90% RH, and 20 ppm, respectively. The ripening time ranged from 0 to 70 h for NDM and MHC and from 0 to 60 h for “Kent” mangos. The color of the samples changed from light green-yellow to gold-yellow in the cases of NDM and MHC, while the green peel color of “Kent” remained the same throughout the ripening period. Each batch of ripening NDM and MHC mangos was evaluated every 12 h. Duplicate measurements of the peel and pulp color on each side of several samples, all at a first glance of a comparable ripening stage, were taken simultaneously. MHC fruits were measured in batches of five mangos. Peel and pulp color were then used to determine ripeness. After measuring peel color, the mangos were halved along the seed and cut lengthways in slices for evaluating the pulp color (Figure 1).

### 2.2. Digital Color Measuring System

**Figure 2:**
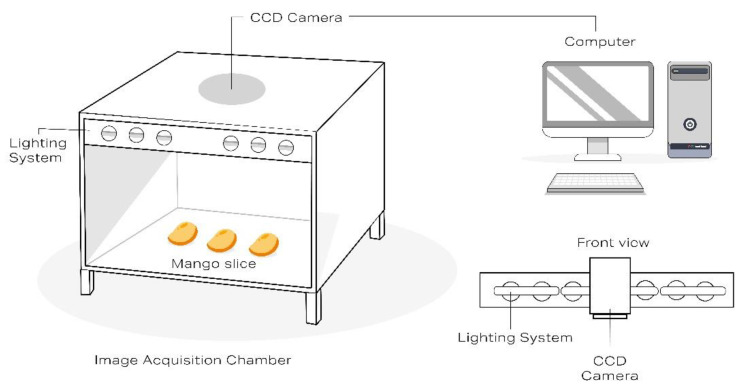
Schematic illustration of the computer vision system setup including the image acquisition chamber for the color measurement of mango slices. CCD: Charge-coupled device camera

The image processing system was supplied by win ing.-Büro Werner Neubauer (Baunach, Germany). The system consists of a CCD camera purchased from IDS Imaging Development Systems GmbH (Obersulm, Germany), a light-tight measuring chamber and a light unit (Figure 2). The camera was connected to a PC via USB interface and controlled by image processing software called WinFoodEval, version 1.21 (win ing -Büro Werner Neubauer, Baunach, Germany). The program was made especially for the evaluation of food. In this case, it was applied to automatically perform the following functions: system calibration, image recording (correction of the brightness), conversion of RGB into HSI, division into color classes, and color evaluation.

The CCD camera had a resolution of 1280 × 1024 pixels, with each pixel having a color resolution of 3 × 8 bits (RGB). The framerate of the camera was 10 frames/s. The lighting system was composed of 16 fluorescent lamps (each lamp 36 W). The light color number was 965, which means it had a color temperature of 6500 K and a color rendering index (CRI) of >90%. A CRI value of 90% or greater (CRI > 90%) is necessary to produce high-quality color images. The lighting chamber dimensions (*w* × *d* × *h*) were 140 cm × 90 cm × 190 cm. A diffusion disk was mounted 50 cm beneath the lamps. The large chamber can generate an even and smooth stream of light in the middle of the measuring area. It was also necessary to maintain the light accuracy required to measure colors in adequate resolution and to meet requirements specified in accordance with the German national standard DIN EN 60350 [26]. The contrast was clearer when the image intensity was not at its maximum setting, i.e., when only half the maximum brightness was used.

The camera was mounted 120 cm above the test samples and inserted into the diffusion disk. The measuring area was 49 cm × 35 cm. Image capturing took place in the closed chamber (Figure 2).

### 2.3. Image Analysis and Color Code Standard

The main task of the WinFoodEval software was to map the measured RGB values from the camera to create a specific color chart designed for mango measurement purposes, including the colors mainly important for mango evaluation. This color chart has three dimensions: one for hue (color), one for chromaticness (ratio of color), and the third one for blackness (ratio of black). However, in order to comply with the NCS, the color chart was converted and labeled with regard to hue (H), chromaticness (S—saturation), and whiteness (I—intensity). It should be noted that this is an adapted HSI color space that is often used in image processing.

**Figure 3:**
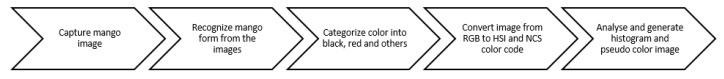
Flowchart for mango color processing using a computer vison system with CCD camera and output. RGB: red, green, and blue color space; HSI: hue, saturation, and intensity color system; NCS: Natural Color Standard.

Figure 3 presents a flowchart briefly illustrating the color measurement process. First, a mango image is captured with the CCD camera. Secondly, the image taken in the previous step is automatically recognized as being a typical mango form. Next, all black and red colors in the image are identified and separated from the other colors, as only the latter are important for mango ripeness evaluation. After that, the image is converted from the camera-originating and -dependent RGB values to HSI values and finally into NCS color codes with the commercially available software WinFoodEval. Finally, a histogram and pseudo-color image are created automatically by the software. In this study, the system’s main function is the color measurement of mango surfaces (peel and pulp). However, it is already used in food industry for other applications (e.g., browning of bread surfaces) [22]. To adapt the system to mangos, it was calibrated in a way that supports the ranges of color, brightness, and saturation that are appropriate for this study.

**Figure 4:**
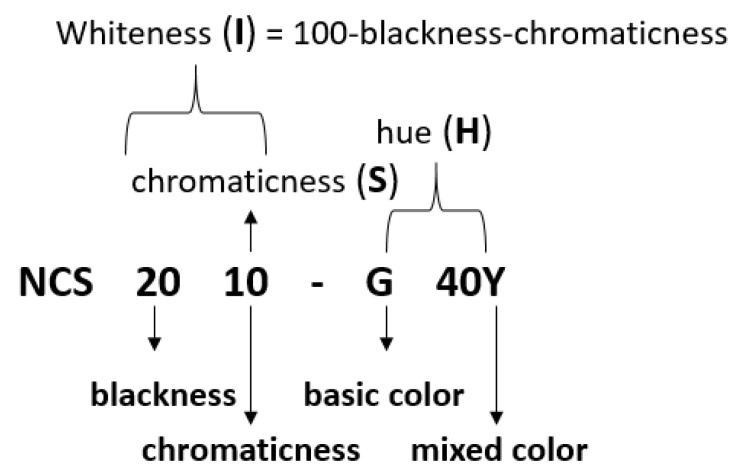
Relation of the Natural Color Standard (NCS) color code with the modified HSI (hue, saturation, and intensity) color system.

After the mathematical conversion of the RGB values, HSI values can be simply transformed to NCS color codes. As shown exemplarily in Figure 4, the color code NCS S 2010-G40Y is denoted by a hue of 60% green basic color and 40% yellow (“G 40Y”). Blackness is at 20% and chromaticness is at 10% (“20 10”). Accordingly, whiteness can be calculated in this example as 70% (100% − 20% − 10% = 70%). This example illustrates a color with weak saturation.

The whole system defines 16 color shades in the range of green (G) to yellow (Y) and red (R). So, final codes can be G, G10Y, G20Y, G30Y, G40Y, G50Y, G60Y, G70Y, G80Y, G90Y, Y, Y10R, Y20R, Y30R, Y40R, Y50R. There can be eight steps in saturation (10, 15, 20, 30, 40, 50, 60, 80) and four steps in brightness (5, 10, 20, 40), leading to a total of 512 color combinations (16 × 8 × 4).

### 2.4. Color Calibration

**Figure 5:**
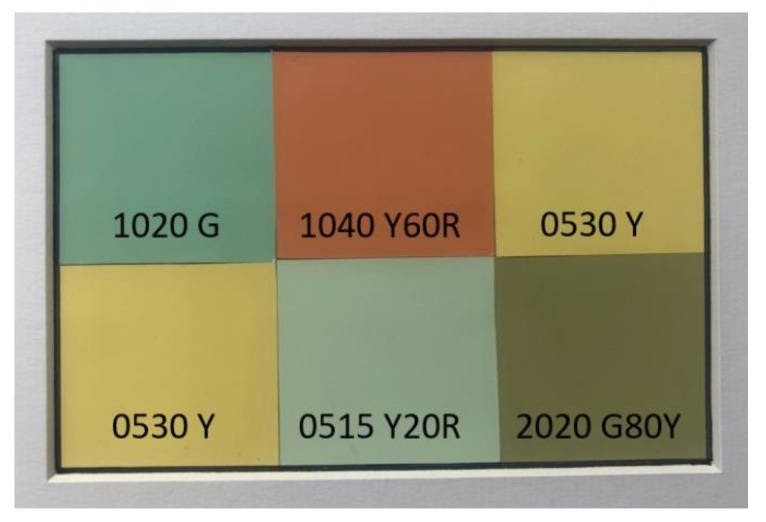
NCS color chart used for the color check for mango evaluation.

The system is initially calibrated using a color chart with an appropriate selection of target colors: ideally, all 512 shade combinations (16 × 4 × 8) have to be calibrated. The calibration also allows for the correction of influencing endogenous factors such as different light sources (e.g., when applying such a system directly in the (mango) field). However, based on the appropriate NCS color chart, which is originally defined by the NCS color standard organization, the most relevant colors typically used for mango evaluation (Figure 5) were applied for a so-called short-check calibration. For that purpose, the camera provides the results as a combination of R, G, and B values. In a very simple way, this color model is cubic and only reflects the color of the sample. Consequently, it needs to be converted to HSI values in order to give information about hue and chromaticness. The conversion of RGB values to HSI values and the integration of the NCS color codes is then calculated by the WinFoodEval software.

For correcting an uneven illumination in the image acquisition chamber, the color calibration was performed using reference standard samples. In this study, the brown color standard with known values of reflected light (similar to intensity) was applied for the system calibration. As specified in the German national standard DIN EN 60350, the calibration model consisted of the 15 color references which are used to calibrate the intensity of the brown color of small cakes. Experience has shown that when the brown colors were used for calibration, the green colors were calibrated automatically. However, the validation of the green colors, commonly known as a short check or color check, was additionally carried out to ensure that the colors were within the range of ±5%. In the following, all results presented were in this range. Consequently, further labeling of standard deviation was omitted.

For this so-called color check, a validated color chart from the Natural Color Standard System^®^ was used as a reference. The color references were selected from colors that are typically included in the mango color range. The following were the five colors required for the color check: S 1020 G, S 1040 Y60R, S 0530 Y, S 0515 G20Y, and S 2020 G80Y. Each block is 4.5 × 4.66 cm (Figure 5) and was directly applied in the chamber of the computer vision system setup. This basic validation was performed before every measurement.

### 2.5. Image Recognition and Color Code Calculation

After the basic calibration, the measurement was performed by placing the mango samples into the image acquisition chamber (Figure 2). The software takes the RGB values from the camera and converts them into color, saturation, and brightness and takes the calibration data into account. This measurement system provides a high-resolution image (1280 × 1024 pixels of color, saturation, and brightness) of the mango samples. All measurement pre-requisites were set in accordance with the German standard DIN EN 60350.

After the software tool had recognized every single mango/mango slice, it automatically searched for colors that were not in the defined color range. Out-of-range colors that are too dark are represented by black spots or red colors.

**Figure 6:**
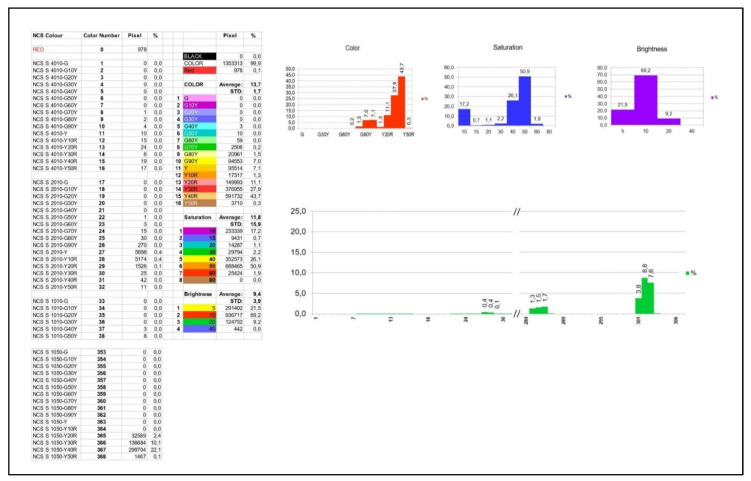
Exemplary data file as provided by the software WinFoodEval.

**Figure 7:**
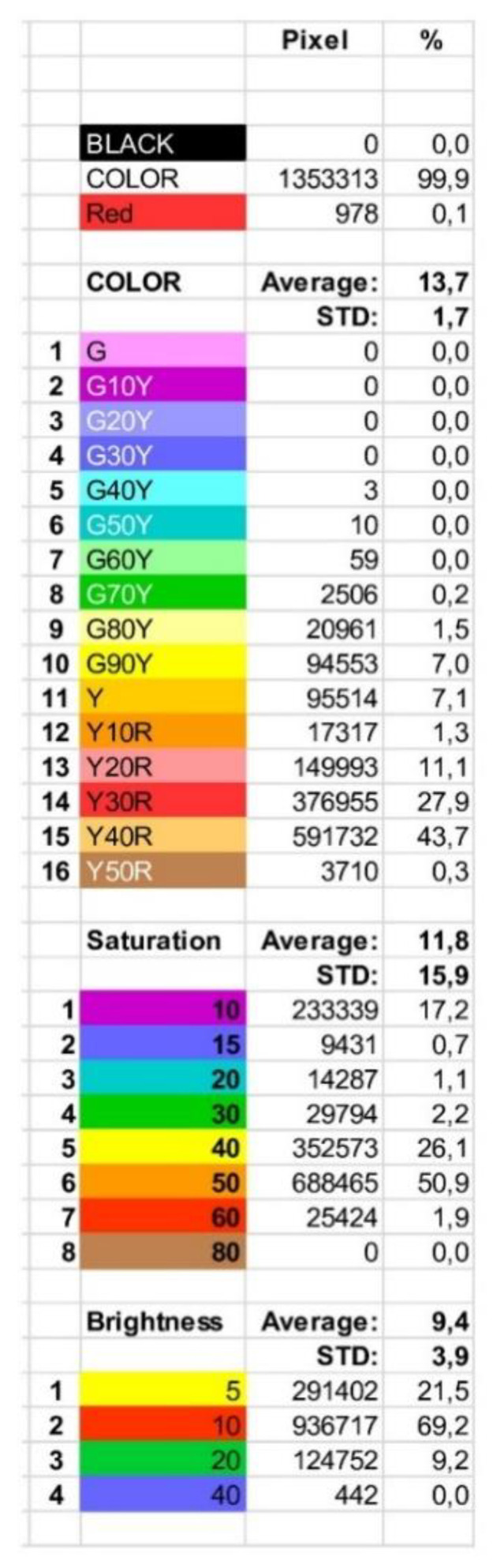
Mango color results as provided by the software WinFoodEval. List of color tones and codes according to the HSI classification. (This is a zoom from Figure 6).

The remaining colors (Figure 6 and Figure 7, denoted as COLOR) were used for conversion into the HSI color space. The relationship between RGB and HSI values was calculated in the following way: more than one hundred RGB values, which served as the reference, were obtained from the color measurement when using the CCD camera. After plotting these values, all of them exhibited a nonlinear behavior. To get the HSI values, a linear interpolation was applied to calculate the HSI value between two points from the nonlinear curve.

The results were expressed as a full *.xlsx data file as exemplarily shown in Figure 6. As this date file is very complex, it is explained in more detail: the pixel values and distribution of black, red, and remaining colors are displayed at the top, respectively (Figure 7). As already explained above, all black and red colors in the image are identified and separated from the other colors, as only the latter are important for mango ripeness evaluation. All pixel values (Figure 7, PIXEL) are calculated as a percentage (%) of the total mango surface.

The next dataset to be interpreted is a list of the colors (Figure 7). As mentioned above in the description of the color transformation, there can be 16 shades in the range of green to yellow (Figure 7, COLOR), eight steps in saturation (Figure 7, SATURATION), and four steps in brightness (Figure 7, BRIGHTNESS).

**Figure 8:**
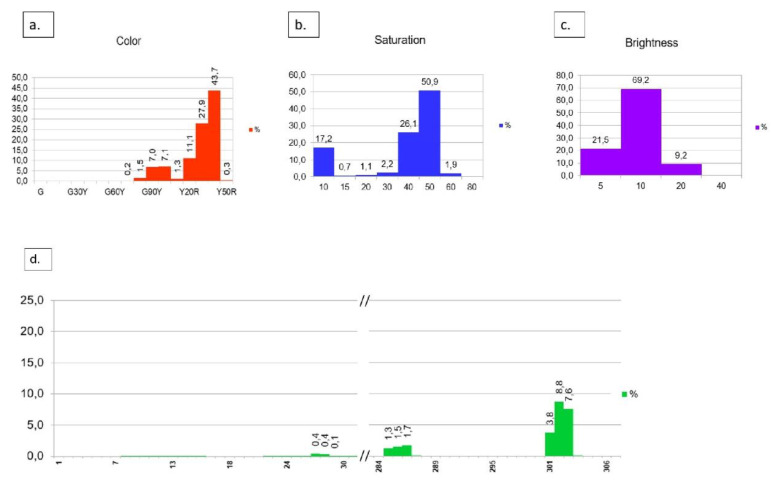
Mango color results as provided by the software *WinFoodEval* as histograms, (**a**) color, (**b**) saturation, (**c**) brightness according to the HSI classification, (**d**) histogram of color codes according to NCS classification. All pixel values were calculated as a percentage (%) of the total mango surface. (This is a zoom from Figure 6).

The pixel percentages for COLOR, SATURATION, and BRIGHTNESS (Figure 7) of an image are further displayed in the form of histograms (Figure 8a–c). The histograms used in this study were described as follows.

- COLOR histogram (16 steps) (Figure 8a),
- SATURATION histogram (eight steps), (Figure 8b),
- BRIGHTNESS histogram (four steps) (Figure 8c).

A fourth histogram illustrates all relevant NCS color code numbers resulting from a measurement (maximally 512 colors) (Figure 8d). The X-axis represents the color number abbreviated from the NCS color code, while the Y-axis shows the percentage of pixels. For visualization purposes, not every color code is presented here, i.e., only relevant code numbers are displayed (Figure 8d).

**Figure 9:**
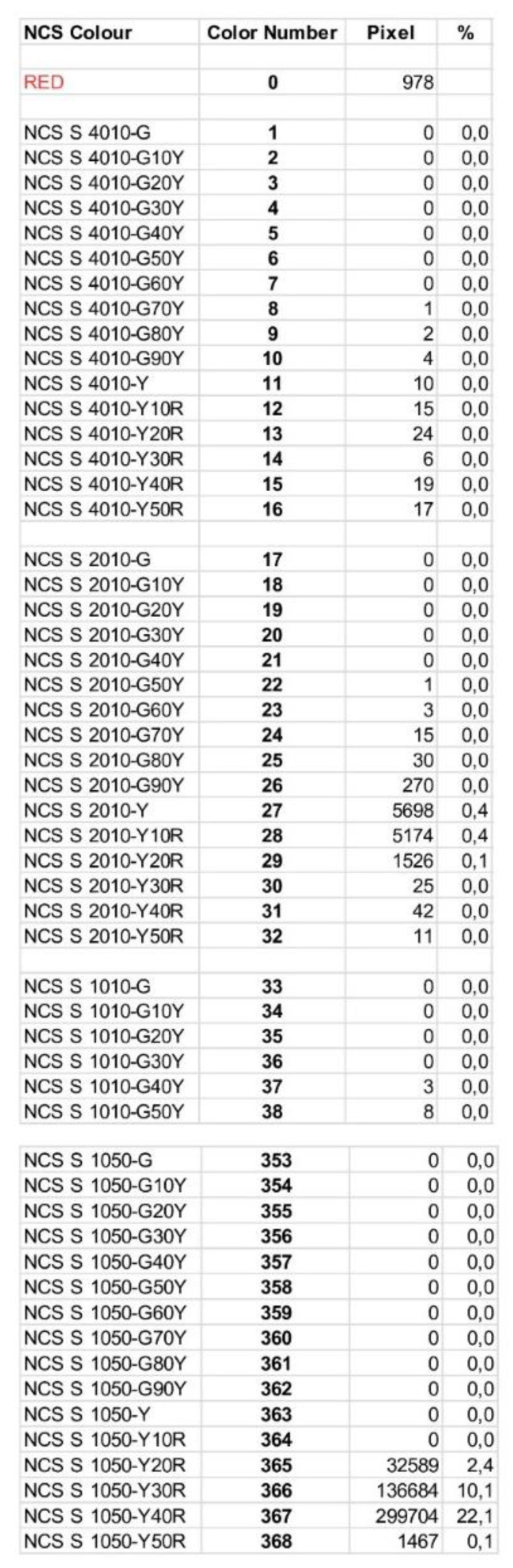
Mango color results as provided by the software WinFoodEval. Color codes according to NCS classification. All codes are given with full abbreviations, pixel values, and distribution. (This is a zoom from Figure 6).

The relevant code numbers are listed in Figure 9. All codes are given with full abbreviations, pixel values, and distribution.

Taking all information together (exemplarily shown in Figure 8a–c), the color providing the highest hue, saturation, and brightness was 43.7%, 50.9%, 69.2%, resulting in the color code Y40R (H), 50 (S), and 10 (I), respectively. Consequently, the color code of this exemplary sample can be labeled as the NCS color code 1050-Y40R. This corresponds to color number 367 (Figure 9) with its value being 22.7% of the complete measured mango area.

Pseudo-color or false color images are also generated in addition to the data file(s). A pseudo-color image represents the mango’s surface as the 16 color codes (Figure 6). The pseudo-color images of this study are shown in Supplementary Materials (Figures S1–S6).

### 2.6. Data Analysis

The comparison of ripeness between different mango cultivars is hardly possible, because color formation is cultivar dependent. Some cultivars remain green, while others change from green to yellow during maturation. Therefore, the maturation progress of the individual mango cultivars was evaluated in this study. As mentioned above, a color check was carried out to ensure that the color determination is within the range of ±5%. Values higher than 5% indicate a shift of the pixel value of a color.

## 3. Results

**Figure 10:**
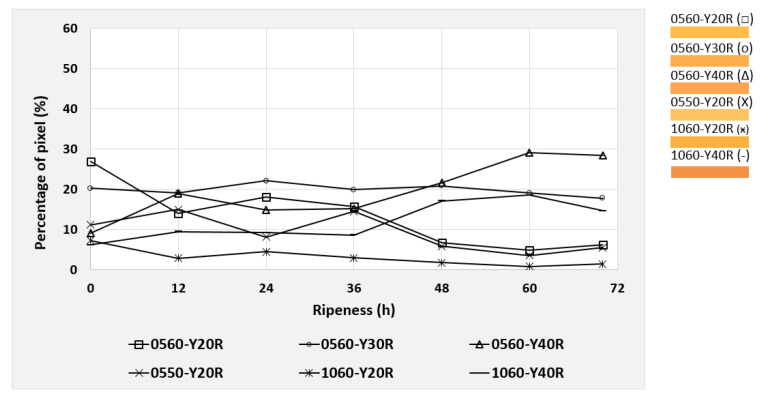
Development of the six most dominating pulp colors of cv. “Nam Dokmai” mangos during the 70 h post-ripening process, represented as NCS color codes.

**Figure 11:**
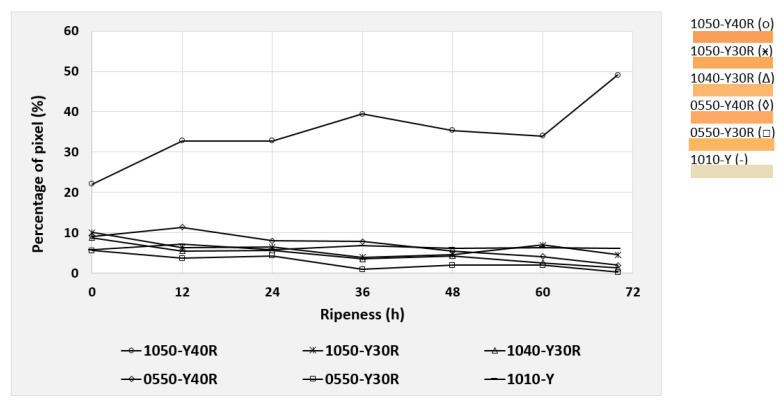
Development of the six most dominating peel colors of cv. “Nam Dokmai” mangos during the 70 h post-ripening process, presented as NCS color codes.

**Figure 12:**
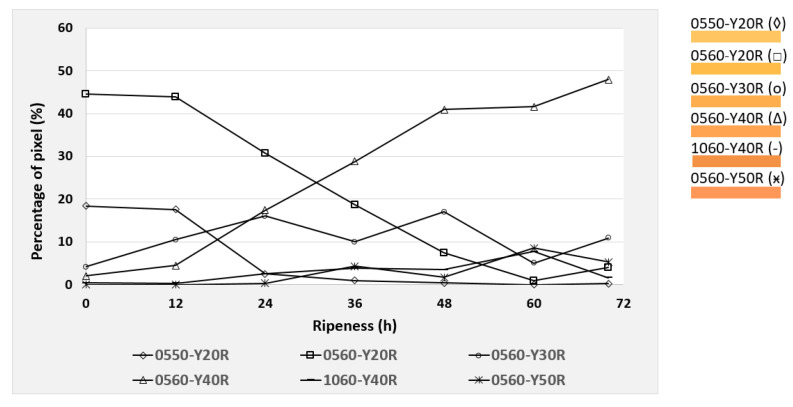
Development of the six most dominating pulp colors of cv. “Mahachanok” mangos during the 70 h post-ripening process, depicted as NCS color codes.

**Figure 13:**
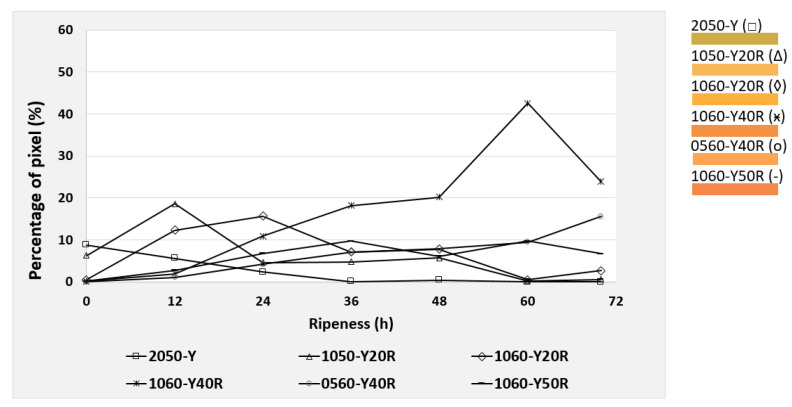
Development of the six most dominating peel colors of cv. “Mahachanok” mangos during the 70 h post-ripening process, depicted as NCS color codes.

**Figure 14:**
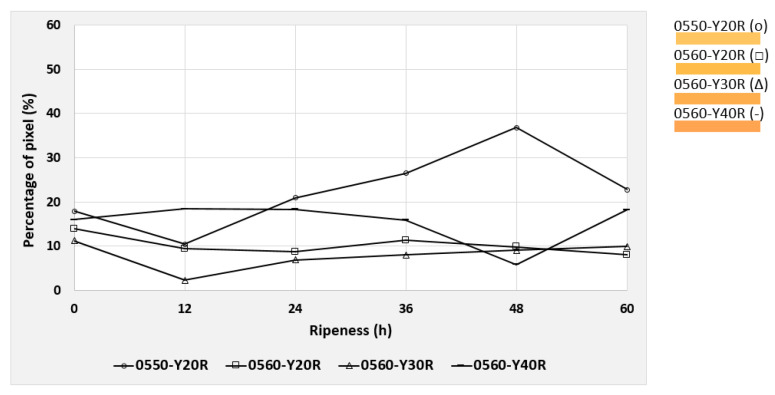
Development of the four most dominating pulp colors of cv. “Kent” mangos during the 70 h post-ripening process, depicted as NCS color codes.

**Figure 15:**
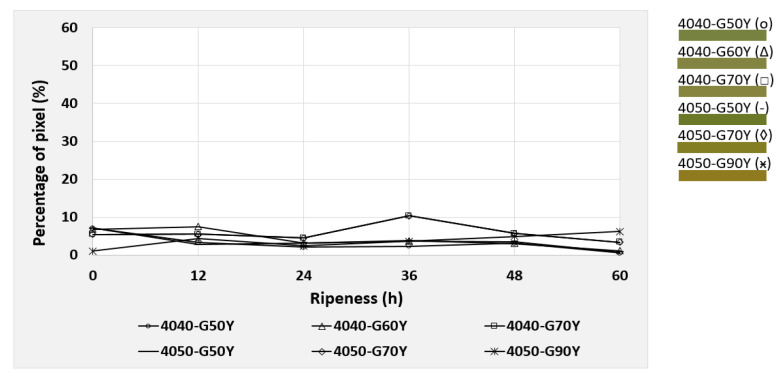
Development of the six most dominating peel colors of cv. “Kent” mangos during the 70 h post-ripening process, depicted as NCS color codes.

Based on the NCS color code calculation described in Section 2.5, the six most dominant color codes were identified and selected for the mango evaluation. These colors were observed during the post-ripening process of the mangos, taking 70 h for cv. “Nam Dokmai” (NDM) and cv. “Mahachanok” (MHC), and 60 h for cv. “Kent”. Each type of mango was monitored in two separate images for peel and pulp color. The results are displayed in the following figures (Figure 10, Figure 11, Figure 12, Figure 13, Figure 14 and Figure 15) and were plotted in two dimensions, in which the X-axis presents the ripening duration up to 70 h and the Y-axis is the percentage of pixels of the corresponding color codes. The six most dominant color codes (with their color) are displayed as a legend on the right side.

### 3.1. Color Determination of Mangos from the cv. “Nam Dokmai” and Development during Post-Ripening

Initially, pulp colors of NDM were evaluated. As shown in Figure 10, most pulp colors of NDM clustered in a small range of pixels. Consequently, those values fall within the calibration of ±5%. However, colors 0560-Y20R and 0560-Y40R changed significantly, due to the changes during the ripening process. These two colors had relatively large values at the beginning and at the end of the ripening process compared to the remaining colors. The progress of the maturation process of the mangos can be determined from the increase in the percentage pixel value of a characteristic color. It was found that after half of the ripening period (36 h), the previously dominant color code was gradually reduced in favor of another code, which individually can be associated with increasing ripeness (for NDM: yellow). However, in the case of any transition of a color code, only the value of percentage pixel was used for the maturity index.

The color 0560-Y20R, consisting of 20% redness and 80% yellowness, decreased with a longer ripening time from 27% to 4.9% of total pixels. The redness increased and the overall shade became 0560-Y30R and gradually changed to 0560-Y40R. There were increasing values of 0560-Y40R from 9.2% to 29.1% with longer ripening time. This color contains 40% redness and 60% yellowness. As a result, the redness increased and the entire shade shifted close to an orange-yellowish tone.

After pulp color evaluation, the peel color of NDM was analyzed. Figure 11 presents the changes of peel colors of NDM taking place during the post-ripening process. Here, only the color 1050-Y40R, which has a redness:yellowness ratio of 40:60, showed a continuously increased pixel intensity from 22.1% to 49.1%. The remaining colors showed only slight differences and can almost be neglected. As the color code has a close relation of yellow and red colors, an increase in its pixel intensity by 27% resulted in the equal combination of redness and yellowness, i.e., 1050-Y40R (representing an orange tone).

### 3.2. Color Determination of Mangos from the cv. “Mahachanok” and Color Development during Post-Ripening

Similarly to NDM mangos, the maturity of MHC pulp can be also characterized by the colors 0560-Y20R and 0560-Y40R, as shown in Figure 12. The first one dropped from 44.5% to 4%, whereas the latter increased from 2% to 47.9%. Principally, these results were similar to those of the NDM mangos, but with larger values for the increase in and reduction of pixel intensity. As a consequence, redness of the code Y20R became Y30R, meaning a total increase in redness by 10%. Furthermore, redness continuously increased to Y40R, as a result of the already mentioned pixel intensity increase from 2% to 47.9%. Color 0560-Y40R consists of 40% redness and 60% yellowness. Accordingly, the pulp shade of MHC turned into an orange-yellowish tone.

Figure 12 and Figure 13 show the pulp and peel color of MHC during the post-ripening process. Obviously, it can be seen that the color 1060-Y40R increased from 0.2% to 42.6% after 60 h and then further decreased to 23.9% afterwards. As its value changed remarkably compared to other colors, it mainly determines peel color. Despite the large fluctuation, the peak shows the increase in pixel intensity being equivalent to the color Y50R. It can be interpreted that the reduction of yellowness dropped from 60% to 50%. Therefore, such a reduction implies the transition into orange shades.

### 3.3. Color Determination of Mangos from the cv. “Kent” and Development during Post-Ripening

The pulp of cv. “Kent” also provided a close similarity in color tones to the other cultivars. As illustrated in Figure 14, the color code 0550-Y20R, consisting of 20% redness and 80% yellowness, increased continuously from 17.9% to 36.8% during the first 48 h and then dropped gradually to 22.7% afterwards. Hence, it is relevant for the determination of the key pulp color of cv. “Kent”. The color code 0560-Y40R decreased and 0560-Y40R was reduced to 0550-Y20R. This illustrated a decrease in redness and an increase in yellowness from 60% to 80%, leading to a more yellowish tone. The remaining colors underwent only slight changes and can be therefore almost neglected.

In contrast to the other cultivars, the peel color tone of “Kent” is quite different, as it is dominated by green colors (Figure 15). However, those six dominating colors remained unchanged and had no distinction in the ripening process from 0 to 60 h. It can be concluded that this method could hardly detect the differences of “Kent” mango peel color, as color change is not that intense without changing the tone significantly.

## 4. Discussion

Color is one of the key sensory characteristics in the evaluation of the quality of mangos and both the surface and the pulp color can be used to assess the degree of ripeness of the fruit. However, color changes, detectable with the naked eye or simple camera systems, are often not very meaningful due to the high similarity between colors in simple color spaces. Furthermore, with simple, camera-based systems, only individual points on the surface can be captured.

Modern computer vision systems enable the inspection of entire fruits on a pixel-based level. Each pixel can be considered as a sample of an original image; more samples typically provide more accurate representations of the original. Due to the tri-chromatic theory, all colors that humans can recognize as an image are a combination of the so-called primary colors red, green, and blue. When digitally captured, pixel-based RGB values are converted into a suitable color model and the corresponding color of an item can be read directly.

A color model is the specification of a three-dimensional coordinate system and of one subspace of this system in which each color is represented by a unique point. When this model is associated with a precise description of how the components are to be interpreted (viewing conditions, etc.), the resulting set of colors is called “color space”. The goal of a color model is to facilitate the specification of colors in a standardized way. However, the choice of a suitable space for color representation remains a challenge for scientists researching color image processing. Blasco et al. (2007) studied the automatic external defect detection of citrus fruit, e.g., oranges and mandarins, using an image processing technique. In total, 635 fruits were tested based on an HSI color space. In that study, they compared five color spaces for the assessment of external fruit defects. As a result, they concluded that the HSI system was highly appropriate for detecting the defects [24]. Similarly, Abdullah et al. (2006) also used the HSI color space for automatic grading of starfruits by color and shape using a computer vision system. They successfully developed a method for categorizing the fruit into different maturity levels. In that study, RGB values were also converted into an HSI system, but the H component was mainly used to classify starfruit into four maturity categories (unripe, underripe, ripe, overripe) [25].

The Natural Color Standard System^®^ has continuously been the international color standard most commonly used in the food industry. Unfortunately, there is a lack of literature describing the application of the NCS for evaluating mango ripeness. Instead, only color systems such as hue, saturation, brightness (HSB) [29], HSI [15,24,30], and CIE L*a*b* [4,17,31] are described and employed for the ripeness measurement of mangos and some other fruits. The present study is one of the first to apply the internationally recognized NCS code to fresh fruits and thus forms the basis for subsequent work in this area.

For the individual valuation of the maturity of different mango cultivars, characteristic values for assessing the color change of the surface and pulp must be determined. By plotting the results in a way that allows for three different colors to be assessed, the color changes that characterize ripening can be determined objectively. These color values can then be integrated into a multifactorial quality and maturity index that allows for an objective assessment of the ripeness of the fruits. It has to be noted in this context, that the maturity index does not depend solely on the fruit color. In addition, firmness, acidity, and total soluble solid, for instance, dry matter, are also used to evaluate fruit maturity [32].

Such an index is particularly useful for optimal post-harvest treatment, as the degree of ripeness of tree-ripened mangos can differ significantly depending on the position of the fruit on the tree and other influencing factors. A suitable sorting and grouping of the fruits, based on their maturity level, are therefore crucial for optimal post-ripening of the fruits [28].

Kienzle et al. (2012) studied the maturity of NDM mango using five parameters, such as titratable acidity (TA), CIE hue angle of the mesocarp, chlorophyll b content, total soluble solids (TSSs), and dry matter (DM), to detect the ripeness. Principle component and cluster analysis were used for data analysis. The results showed that TA and hue angle were the two most significant attributes describing maturity of NDM, followed by chlorophyll b and TSSs [33]. Lebrun et al. (2008) determined the volatiles of mango cultivars “Cogshall”, “Kent”, and “Keitt” using an electronic nose and gas chromatography in order to differentiate the harvest maturities and ripening stages in different sizes. Moreover, the effect of harvest maturity on mango flavor was studied. The use of an e-nose approach was able to distinguish among the volatiles of the five size categories of “Keitt” mangos, while “Kent” mangos were categorized between three of the five sizes. In addition, the e-nose could also differentiate between the volatiles of ripe “Kent” and “Keitt” mangos [32]. Suwansichon et al. (2012) analyzed nine mango cultivars, including NDM, by means of sensory evaluation for determining the stage of ripeness. Twenty flavor attributes and eleven texture attributes were evaluated by a trained panel. The results showed that the attributes could be used to describe the variations in the degree of ripeness in terms of both taste and texture characteristics between the samples. [34]. However, all these methods are invasive and need trained sensory panelists for performing the analyses.

The assessment of the surface color offers various advantages in this context: it is a non-invasive method that can be carried out immediately after the harvest in order to make an initial assessment of the degree of ripeness. As in other sensory examinations, the surface color is traditionally assessed by appropriately trained and experienced panelists. However, these methods can sometimes be very error-prone: In addition to the observers’ individual assessment standards, there are also external factors such as time of day, incidence of light, and light intensity that can influence the color assessment. Furthermore, the significance of conventional imaging inspection methods, such as colorimetric or spectrophotometric procedures, is limited, since only a few individual measuring points can be recorded. Khairunniza-Bejo et al. (2014) reviewed the limitation of conventional color measuring methods with a colorimeter and spectrophotometer. The authors concluded that such methods only give a single average reading over only one spot of a sample, but not all surface pixels available. Further, the use of these methods showed, due to low spatial resolution, a limited color sensing capacity. In addition, some approaches are not applicable with non-homogeneous color [1].

In contrast, modern automated computer vision systems may allow for a standardized and reproducible assessment of the surface color and might have the potential to be used in commercial industry as a rapid test method for fruit sorting. The present study showed that the use of CCD cameras could successfully be applied for evaluating the color and the ripeness of mangos. More specifically, the observed changes in pixel intensity according to the commonly applied color codes can indicate the degree of fruit ripeness. The peel color of NDM and MHC differed slightly. NDM can be described by the color 1050-Y40R, while MHC is mainly characterized by 1060-Y40R. They have distinct saturation levels of 50% and 60%, respectively. Vásquez-Caicedo et al. (2004) noted that the peel color of NDM did not really change during ripening at room temperature, when measuring with a colorimeter [35]. In the present study, however, changes in peel color of NDM were observed, when using a computer vision system under defined ripening conditions. It should be noted that the results of the present experiment were also based on a calibration system which used the color green (consisting of five different tones) as a reference with a tolerance range set to ±5%. Therefore, the measured data were always in the acceptable range, indicating a valid and accurate measurement. 

The codes 0560-Y20R and 0560-Y40R were the two most relevant codes for observing the maturity of both NDM and MHC fruits with regard to their pulp colors. The pixel intensity reduction of the code 0560-Y20R, which has 20% redness and 80% yellowness, means that the yellow color is reduced within the yellow-orange zone, which ultimately indicates the ripeness. Similarly, an increase in the presence of the code 0560-Y40R (40% redness and 60% yellowness) also indicated ripeness in both NDM and MHC mangos. Vásquez-Caicedo et al. (2005) found that, due to the accumulation of β-carotene, the pulp of NDM and MHC developed a yellow-orange color. The same pattern was also found in the present study [36]. Nandi et al. (2012) also discovered similar results in which the same color pattern was observed at different levels of ripeness, particularly along the fruit’s apex region [16].

Based on the abovementioned results, the computer vision system has proven to be a suitable methodology for estimating the pulp and peel colors of the mango cv. “Nam Dokmai” and “Mahachanok”, as well as the pulp colors of cv. “Kent”. Peel color changes of cv. “Kent” could not be assessed because the fruits kept their rich green color during the entire post-ripening period and developed no detectable color gradients. Sivakumar et al. (2011) also postulated that skin color is not always applicable for evaluating the maturity index of mangos that have a green surface color even when fully mature [37]. The restrictions when assessing the surface color must also be considered when assessing the ripeness of other fruits, especially those with a high proportion of green in the skin color. In addition, only fruits that show a significant change in color during ripening can be assessed on the basis of the surface color. 

Inevitably, there are inconsistencies in fruit maturity. Mangos at different stages of ripening can lead to significant measurement fluctuations. Consequently, the present study tried to minimize the interindividual fruit maturity status by a parallel measurement of at least several samples of a batch in parallel. At the beginning of the experiments with MHC, five samples were measured (Figures S1 and S2). During the experiments, it was realized that three samples might already be enough for evaluating the other mango varieties. Such a number should be used to represent a full sample of each ripening hour for compensating color fluctuations. In order to identify peel colors more accurately, the same fruit sample should be measured throughout the whole duration of the ripening period. However, this is not applicable for pulp color measurement, as it must be cut for the measurements and cannot be measured again afterwards.

## 5. Conclusions

This study focused on the automated color measurement of mango peel and pulp using a computer vision system and color standards. Namely, “Nam Dokmai”, “Mahachanok”, and “Kent” mango varieties were investigated in this study. Peel and pulp colors represent the change in pigments that occurs during mango ripening and can therefore be used to identify different levels of mango ripeness. A combined method using both a computer vision system and the color-coded index can precisely predict the ripeness stage of mangos with a color pixel range precision of ± 5%. However, the method seems to be less suitable for samples with no gradient color change over different maturity stages (in this case: cv. “Kent” (peel)). 

Correlations with the traditional parameters for evaluating ripeness, such as firmness, acidity, total soluble solids, and further parameters, have to be made in future studies to get a clearer view of mango quality and biochemical mechanisms behind post-ripening processes during transport and storage in stores, shops, or under household conditions. 

## Supplementary Materials

The following are available online at https://www.mdpi.com/2304-8158/9/11/1709/s1, Figure S1: Peel color and pseudo-color images of “Mahachanok” (MHC) mango depending on ripening duration; Figure S2: Pulp color and pseudo-color images of “Mahachanok” (MHC) mango depending on ripening duration; Figure S3: Peel color and pseudo-color images of “Nam Dokmai” (NDM) mango depending on ripening duration; Figure S4: Pulp color and pseudo-color images of “Nam Dokmai” (NDM) mango depending on ripening duration; Figure S5: Peel color and pseudo-color images of “Kent” mango depending on ripening duration; Figure S6: Pulp color and pseudo-color images of “Kent” mango depending on ripening duration.
