# Supplementary figures and images for: Developing an Automatic Color Determination Procedure for the Quality Assessment of Mangos (Mangifera indica) Using a CCD Camera and Color Standards
Source: Foods. 2020 Nov 21;9(11):1709. doi: 10.3390/foods9111709 (PMC7700315; doi:10.3390/foods9111709)

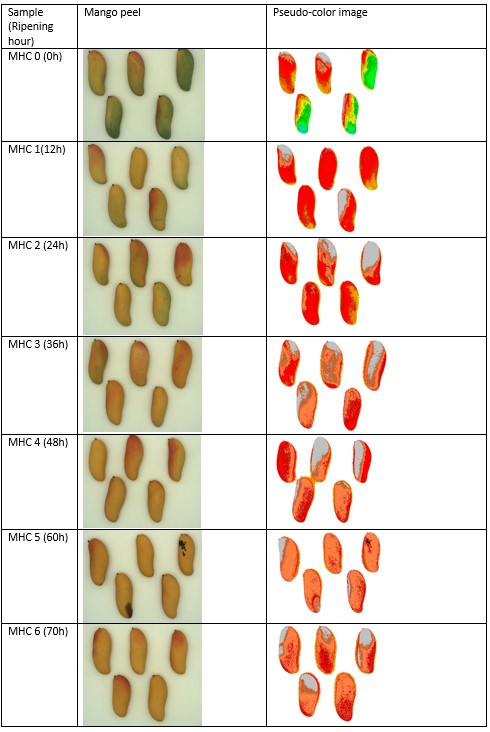

Supplement: Supplementary file 1 [file foods-09-01709-s001.zip › Figure A1.jpg]

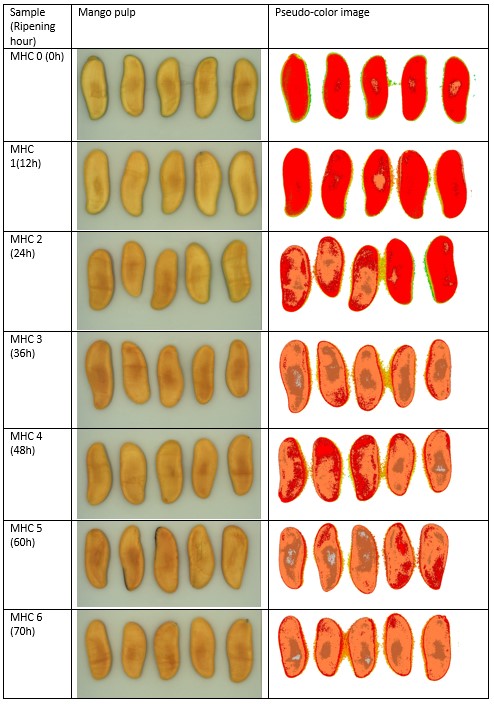

Supplement: Supplementary file 1 [file foods-09-01709-s001.zip › Figure A2.jpg]

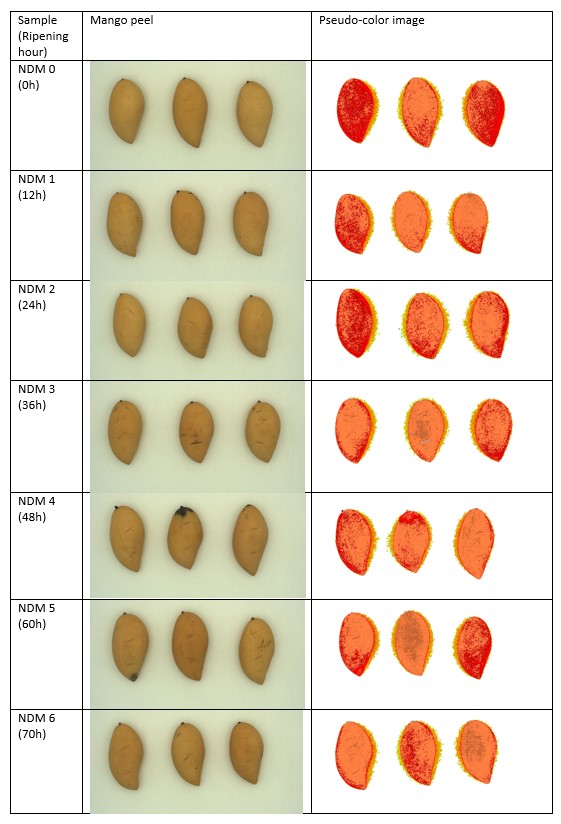

Supplement: Supplementary file 1 [file foods-09-01709-s001.zip › Figure A3.jpg]

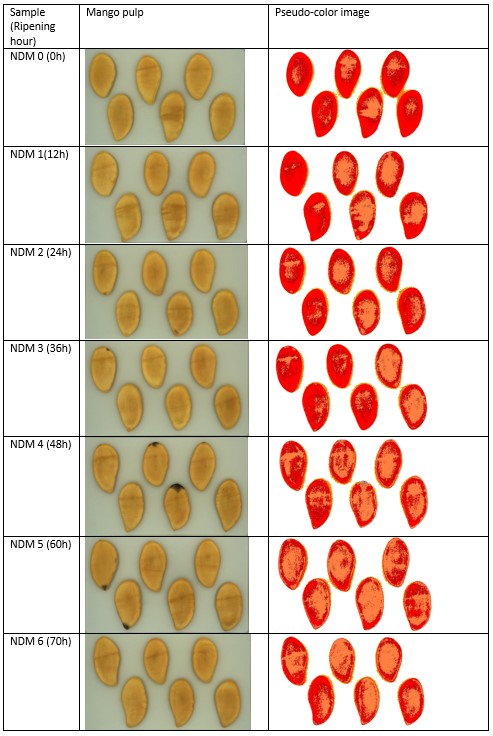

Supplement: Supplementary file 1 [file foods-09-01709-s001.zip › Figure A4.jpg]

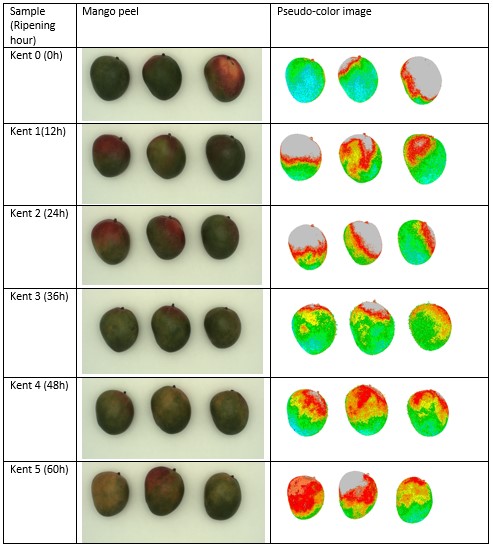

Supplement: Supplementary file 1 [file foods-09-01709-s001.zip › Figure A5.jpg]

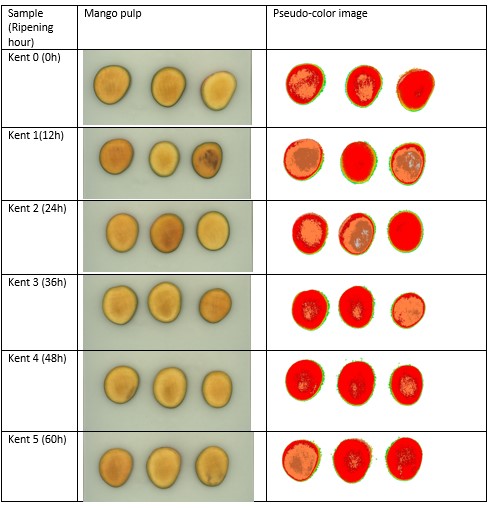

Supplement: Supplementary file 1 [file foods-09-01709-s001.zip › Figure A6.jpg]
